# Entrepreneurship Education and Entrepreneurial Intentions of College Students: The Mediating Role of Entrepreneurial Self-Efficacy and the Moderating Role of Entrepreneurial Competition Experience

**DOI:** 10.3389/fpsyg.2021.727826

**Published:** 2022-01-06

**Authors:** Lihao Wu, Suo Jiang, Xiaomin Wang, Linwei Yu, Yansu Wang, Hui Pan

**Affiliations:** ^1^School of Foreign Languages Studies, Wenzhou Medical University, Wenzhou, China; ^2^Department of Applied Psychology in School of Psychiatry, Wenzhou Medical University, Wenzhou, China; ^3^School of Innovation and Entrepreneurship, Wenzhou Medical University, Wenzhou, China

**Keywords:** entrepreneurship education, entrepreneurial self-efficacy, entrepreneurial intention, mediating role, moderating role

## Abstract

This study aims to explore effective ways to improve college students’ entrepreneurial self-efficacy and intentions through entrepreneurship education. The survey used a random sample of 804 college students in Zhejiang Province, China. The results show that: (1) In terms of the characteristics of entrepreneurial intention, there are significant differences in gender, entrepreneurial experience, entrepreneurial competition experience, and family background of self-employment. (2) There are significant differences in the characteristics of entrepreneurship education in gender, entrepreneurial competition experience, and the family background of self-employment. (3) In the relationship among entrepreneurship education, entrepreneurial self-efficacy, and entrepreneurial intention, entrepreneurship education is significantly and positively related to entrepreneurial self-efficacy and entrepreneurial intention. Entrepreneurial self-efficacy is significantly and positively associated with entrepreneurial intention. Entrepreneurial self-efficacy plays a complete mediating role between entrepreneurship education and entrepreneurial intention. Entrepreneurial self-efficacy also has a suppressing effect on the relationship between the two. (4) Entrepreneurial competition experience moderates the second half of the mediating effect of entrepreneurial self-efficacy. Finally, the study offers several proposals for the teaching practice of entrepreneurship education.

## Introduction

Entrepreneurship is the core power of growing economy in a country or region (Van Praag and Versloot, 2007). Entrepreneurship can promote economic development, industrial upgrading, and the transformation of economic structure as well as creating jobs, thus pushing social progress forward (Hathaway and Litan, 2014). In the field of entrepreneurship, researchers believe that entrepreneurial behavior does not result from entrepreneurial business opportunities directly, and entrepreneurial intentions lie behind entrepreneurial behavior (Krueger, 2007). Entrepreneurial intention refers to the belief that an individual intends to start a new company and consciously plans to put it into practice at some point in the future (Thompson, 2009). It can significantly predict entrepreneurial behavior (Krueger et al., 2000). Entrepreneurial self-efficacy is also a very important explanatory variable when determining the intensity of entrepreneurial intentions and the likelihood that these intentions will lead to entrepreneurial behavior. Present studies have shown that entrepreneurial self-efficacy is a key indicator for effective prediction of college students’ entrepreneurial intentions and entrepreneurial behavior, and also an important prerequisite for potential entrepreneurs to initiate entrepreneurial behavior (Krueger et al., 2000). In fact, many studies on entrepreneurial intention prediction have chosen to use the variable of entrepreneurial self-efficacy (Krueger et al., 2000; Van Gelderen et al., 2006; Liñán et al., 2011; Lortie and Castogiovanni, 2015; Mei et al., 2020). Entrepreneurship education is likely to enhance individuals’ entrepreneurial intentions by improving individuals’ entrepreneurial self-efficacy.

Entrepreneurship education plays an irreplaceable role in improving college students’ entrepreneurial intentions, promoting college students’ employment and entrepreneurship, and boosting economy. Empirical research shows that college students stand better chances to succeed in entrepreneurship and have better prospects for business development (Shane, 2004). By turning knowledge into social value, college students’ entrepreneurship has become one of the driving forces of socio-economic development (Shane, 2004). In recent years, in order to promote college students’ entrepreneurship, universities have generally launched entrepreneurship education, and the cultivation of entrepreneurial skills has been regarded as one of the main issues of higher education. Entrepreneurship education helps college students establish positive career concept and professional ideals, improve their comprehensive qualities and practical ability, develop creative thinking, and promote college students to actively participate in social competition. However, a few scholars explore the mechanism of entrepreneurial self-efficacy as a mediator and entrepreneurial competition experience as a moderator in depth in the link between entrepreneurship education and entrepreneurial intention in the context of China. Although some western researchers have begun to pay attention to the important role of prior experience to explain entrepreneurial intention and firm performance (Miralles et al., 2016; Sarma and Marszalek, 2020; Jiao et al., 2021), only a few researchers have explored the role of entrepreneurial competition experience in the study of entrepreneurship (Schwartz et al., 2013; Yu, 2013; Brentnall et al., 2018). China’s innovation and entrepreneurship education research emerged in the late 1990s, which is much later than that in the west. To date, a few scholars have examined the moderating effect of entrepreneurial competition experience.

Therefore, this study intends to investigate the characteristic of college students’ entrepreneurial intention and entrepreneurship education, the relationship between them, as well as the mediating role of entrepreneurial self-efficacy and the moderating role of entrepreneurial competition experience, in order to explore effective ways to enhance college students’ entrepreneurial intention through entrepreneurship education.

## Theories and Assumptions

### Entrepreneurship Education and Entrepreneurial Intention

Entrepreneurship education is an educational activity that enhances students’ entrepreneurial knowledge, skills, attitudes, and personal qualities (Liñán et al., 2008). Entrepreneurship education is also defined as an entrepreneurship education course offered by universities that teaches the theory and practice of entrepreneurship (Ginanjar, 2016). Entrepreneurship education in universities is in a unique position, not only influencing and shaping students’ attitudes toward entrepreneurship, but also cultivating students’ entrepreneurial perspectives so that students can play multiple roles in the entrepreneurial process. A longitudinal survey of business students was carried out at a British university and found that the students have higher entrepreneurial learning and inspiration compared to their counterparts without entrepreneurship education (Nabi et al., 2018). Entrepreneurship education had greatly improved college students’ business knowledge and skills, and remarkably increased their involvement in small businesses after graduation (Loy, 2014; Vaughan, 2014; Egan et al., 2017). Many studies show strong correlations between students’ participation in entrepreneurship education and the formation of their entrepreneurial intention (Lüthje and Franke, 2003; Tiwari et al., 2017; Wang et al., 2019).

Thompson (2009) defines an individual’s entrepreneurial intention as “a person intends to establish a new enterprise and consciously plans to do so at some time in the future.” As a state of consciousness before action (Buttar, 2015), entrepreneurial intention plays a key role in the decision of individuals to establish a new business (Bird, 1988; Nabi et al., 2010), and is considered to be the best predictor of entrepreneurial behavior (Krueger et al., 2000; Carsrud and Braennback, 2011). Recently, many researchers have begun to focus on the entrepreneurial intentions of college students (Sanchez, 2013; Støren, 2014; Zhang et al., 2014; Udayanan, 2019). Many factors are considered to be determinants of entrepreneurial intentions, such as personality factors, environmental factors, and population factors (Indarti and Rostiani, 2008). There are still some other factors that affect an individual’s entrepreneurial intentions, such as origin, religious belief, education level, work experience, etc. (Reynolds et al., 1994), which are usually referred to as demographic variables. At the same time, research also found that boys’ entrepreneurial intentions are higher than girls’ (Cohoon et al., 2010; Pines et al., 2010). Faloye and Olatunji (2018) found entrepreneurship education, ability to take risks, family and friends, including mentor, were the significant effects of entrepreneurship intentions among the participants. The firmer an individual’s entrepreneurial intention becomes, the more likely for him/her to have entrepreneurial behavior. To promote students’ entrepreneurship intention, entrepreneurship educators need to provide students with various learning opportunities (Bagheri et al., 2013).

In recent years, researchers have begun to pay more attention to the relationship between entrepreneurship education and entrepreneurial intentions. Many researchers found that the level of entrepreneurship education has a significant impact on individual entrepreneurial intentions, and entrepreneurship education significantly enhanced students’ entrepreneurial intentions (Hou et al., 2019; Mei et al., 2020; Zhang and Huang, 2021), while some studies came to the opposite conclusions that entrepreneurship education failed in promoting students’ entrepreneurial intentions of becoming an entrepreneur (Kusumojanto et al., 2020). Other studies have also shown that participation in entrepreneurship courses and training has a positive impact on students’ entrepreneurial intentions (Souitaris et al., 2007). On this basis, this research proposes the first research hypothesis:

*H1*: Entrepreneurship education is significantly and positively related to entrepreneurial intentions.

### The Mediating Effect of Entrepreneurial Self-Efficacy

American psychologist Bandura (1977) first proposed the concept of “self-efficacy.” He believed that self-efficacy is an individual’s self-assessment and judgment for the completion of a certain behavior. The concept of entrepreneurial self-efficacy is derived from Bandura’s self-efficacy. Chen et al. (1998) gave the definition of “Entrepreneurial self-efficacy,” that is, “the strength of an individual’s belief that he or she is capable of successfully performing the roles and tasks of an entrepreneur.” Entrepreneurship self-efficacy is related to self-confidence, will, and persistence to overcome the initial anxiety caused by the new startup. In 1998, Chen et al. developed the “Entrepreneurship Self-Efficacy Scale” based on previous research, which effectively distinguished entrepreneurs from non-entrepreneurs. The research has been unanimously affirmed by many researchers (Markman and Baron, 2003; Baum and Locke, 2004).

In recent years, researchers pay more attention to entrepreneurial self-efficacy. Many studies indicated that entrepreneurship education positively influenced entrepreneurial self-efficacy (Wilson et al., 2007; Kusumojanto et al., 2020; Wardana et al., 2020), thereby enhancing individual entrepreneurial intentions (Wilson et al., 2007). At the same time, a series of studies have shown that entrepreneurial self-efficacy is a positive mediation between entrepreneurship education and entrepreneurial intentions (Fuller et al., 2018; Mei et al., 2020; Wardana et al., 2020). However, some research showed an insignificant impact between self-efficacy and entrepreneurial intention (Ogunleye and Osagu, 2014; Ferreira et al., 2017). Based on the above description, we believe that entrepreneurial self-efficacy may be a positive mediation between entrepreneurship education and entrepreneurial intentions, so the research hypotheses are established as follows:

*H2*: Entrepreneurship education is significantly and positively related to entrepreneurial self-efficacy.

*H3*: Entrepreneurial self-efficacy is significantly and positively linked to entrepreneurial intentions.

*H4*: Entrepreneurial self-efficacy plays a mediating role in the relationship between entrepreneurship education and entrepreneurial intentions.

### Entrepreneurial Competition Experience as a Moderator

Researchers have explored prior experience of the individual and its relationship with entrepreneurial variables from different perspectives. Social learning theory postulates a direct link between experience and self-efficacy. It has been shown that personal successes reinforce self-efficacy, while failures tend to undermine it (Bandura, 1977; Bandura et al., 2001). Students who have already engaged in entrepreneurial practice have higher entrepreneurial intentions than their counterparts without prior experience (Carr and Sequeira, 2007). From the perspective of human capital theory, research shows that growth aspirations will be higher for individuals with higher educational attainment and lower for those with prior entrepreneurial experience (Capelleras et al., 2019). Jiao et al. (2021) also confirm the significant correlation between prior experience and firm performance by drawing on human capital theory. Some researchers study the impact of prior experience on entrepreneurial intention based on the theory of planned behavior (Miralles et al., 2016). Others pay attention to the moderating role of prior experience on variables, such as human capital, social capital, role models, entrepreneurial self-efficacy, and intention (Shirokova et al., 2015; Brunel et al., 2017; Hernández-Carrión et al., 2017).

As part of prior experience, entrepreneurial competition experience has gained some attention from some researchers. Some research has emphasized on the impact of entrepreneurial competition experience to promote entrepreneurship education. Watson and McGowan (2019) focus on the university-based business plan competition and propose a shift from competition to coopetition-based entrepreneurship education by drawing on the theory of effectuation. Some researchers have investigated how entrepreneurship education can be advanced through capacity building by evaluating an annual teen entrepreneurship competition which has lasted for 8 years (Yu, 2013). Nevertheless, there is only limited knowledge regarding the moderating role of entrepreneurial competition experience in the relationship between entrepreneurship education, entrepreneurial self-efficacy, and intention. As researchers have emphasized the important role of prior experience in explaining entrepreneurial self-efficacy and entrepreneurial intention (Morris et al., 2012; Miralles et al., 2016), we propose our next hypothesis:

*H5*: Entrepreneurial competition experience would moderate the second half of the mediating effect of entrepreneurial self-efficacy. That is to say, the link between entrepreneurial self-efficacy and entrepreneurial intention would be stronger for individuals with high levels of entrepreneurial competition experience.

## Materials and Methods

### Participants and Procedure

The survey was conducted from March to May 2021 in four universities in Wenzhou of Zhejiang Province in China. Wenzhou is considered as the Capital of Entrepreneurship with a long entrepreneurial history and numerous entrepreneurial practice, so Wenzhou is a representative district for the study of entrepreneurship education and intention. Sex, major, grade, and school of the participants were taken into account and a stratified random sampling was implemented. A total of 804 questionnaires were distributed by an online questionnaire survey platform Questionnaire Star. The participants are mainly college students. The researchers sent the questionnaire link to the class counselors *via* WeChat and QQ and asked them to forward it to the class group for students to fill out online. Among them, 379 are in the first year of university (47.1%), 86 are in the second year of university (10.7%), 269 are in the third year of university (33.5%), 61 are in the fourth year of university (7.6%), and 9 (1.1%) have a master’s degree or above. The majors involved included 274 in medicine (34.1%), 147 in science and engineering (18.3.9%), 232 in economics and management (28.9%), 112 (13.9%) in humanities, and 39 (4.9%) in other majors.

### Instruments

The research questionnaire consists of two parts. The first part is the basic information of the participants. In entrepreneurship research, demographic factors and other variables related to past experience are generally used as control variables, which have some influence on the prediction of individual entrepreneurial intentions, such as gender, major, grade, family background, and previous personal entrepreneurial experience. The second part is the measurement of entrepreneurship education, entrepreneurial self-efficacy, entrepreneurial intention, and other variables. In order to make the research results more accurate, the existing scales of the variables referred to in this research are summarized. It is found that the current measurement scale for entrepreneurial self-efficacy has been developed relatively mature. This research mainly draws on the entrepreneurial self-efficacy scale developed by Lucas and Cooper (2005). This scale includes 5 dimensions: innovation efficiency, risk-taking, opportunity identification, relationship coordination, and organizational commitment, with a total of 16 items. Cronbach’s alpha of the entrepreneurial self-efficacy scale is 0.968, the KMO value is 0.964, and the significance of Bartlett’s test of sphericity is 0.000 < 0.05. Entrepreneurship education draws on the Entrepreneurship Education Scale of Franke and Lüthje (2004), which measures entrepreneurship education in two dimensions, one is school factors and the other is personal factors. The scale includes nine questions. Cronbach’s alpha of the entrepreneurship education scale is 0.903, the KMO value is 0.874, and the significance of Bartlett’s test of sphericity is 0.000 < 0.05. The entrepreneurship intention scale is based on previous studies, drawing lessons from Chen et al. (1998) and Fayolle and Liñán (2014) on individual entrepreneurial intention scales, and has been appropriately revised in light of the background of China’s entrepreneurship education. There are seven questions in total. Cronbach’s alpha of the entrepreneurial intention scale is 0.957, the KMO value is 0.899, and the significance of Bartlett’s test of sphericity is 0.000 < 0.05. The reliability coefficients of the three scales are all greater than 0.7, and the KMO value is greater than 0.5. The significance of Bartlett’s test of sphericity is less than 0.05, indicating that the three scales all have high reliability and a good internal consistency structure. Each question on the scale is measured by Likert’s 5-level scoring scale method from “Strongly Disagree” to “Strongly Agree.”

### Data Analysis

SPSS 23.0 was used to conduct descriptive, independent sample *t*-test, one-way ANOVA, correlation, and regression analysis. Hayes (2017) SPSS macro program PROCESS was used to analyze and test the mediating effect. The SPSS macro program can verify various mediated moderation and moderated mediation models based on Bootstrap method of deviation correction percentile. 5,000 Bootstrap samples were extracted to obtain the robust standard error and Bootstrap confidence interval of parameter estimation. If the confidence interval does not include 0, the result is statistically significant. Model 4 and Model 59 in the SPSS macro program were selected to analyze the mediating effect and moderated mediation model in this study. To obtain the standardized regression coefficients, we transformed all the raw scores to z-scores before testing the mediated moderation effect.

## Results

### Descriptive Statistics

A descriptive statistical analysis of gender, grade, major, entrepreneurial experience, entrepreneurial competition experience, and family background of self-employment of entrepreneurial intention is conducted. The results are shown in Supplementary Table 1. In order to further examine the differences of entrepreneurial intention in these variables, independent sample *t*-test and one-way ANOVA are conducted. The independent sample *t*-test found that the measurement of entrepreneurial intention of boys was significantly higher than that of girls, *t*_(802)_ = 4.33, *p* < 0.001. The entrepreneurial intention of students with entrepreneurial experience is significantly higher than that of students without entrepreneurial experience, *t*_(802)_ = 5.85, *p* < 0.001; the entrepreneurial intention of students who have participated in entrepreneurial competitions is significantly higher than that of students who have not participated in entrepreneurial competitions, *t*_(802)_ = 3.78, *p* < 0.001. The entrepreneurial intention of students with family background of entrepreneurship is significantly higher than that of students without it, *t*_(802)_ = 4.17, *p* < 0.001. A one-way ANOVA was performed on grades and majors, and the results showed that there was no significant difference in entrepreneurial intentions between different majors and grades, *p* > 0.05.

Supplementary Table 2 is the descriptive analysis of the gender, grade, major, entrepreneurial experience, entrepreneurial competition experience, and family background of self-employment of entrepreneurial education. In order to further examine the differences in the control variables of entrepreneurship education, independent sample *t*-tests and one-way ANOVA were performed. Independent sample *t*-test results show that in entrepreneurship education, boys’ scores are significantly lower than those of girls’, *t*_(802)_ = −3.17, *p* = 0.002; the scores of students who participated in entrepreneurial competitions were significantly higher than those who did not, *t*_(802)_ = 5.32, *p* < 0.001; the scores of students with family background of entrepreneurship were significantly higher than those without it, *t*_(802)_ = 2.85, *p* = 0.005. There was no significant difference between the scores of students with entrepreneurial experience and those without entrepreneurial experience, *p* > 0.05. A one-way ANOVA found that there was also no significant difference in the scores of entrepreneurship education in different majors and grades, *p* > 0.05.

### Correlation Analysis

The correlation analysis (see Supplementary Table 3) indicates that entrepreneurship education was positively correlated with entrepreneurial intentions and entrepreneurial self-efficacy. There was also a positive correlation between entrepreneurial self-efficacy and entrepreneurial intentions.

### Testing for Mediating Effect and Path Analysis

**Figure 1:**
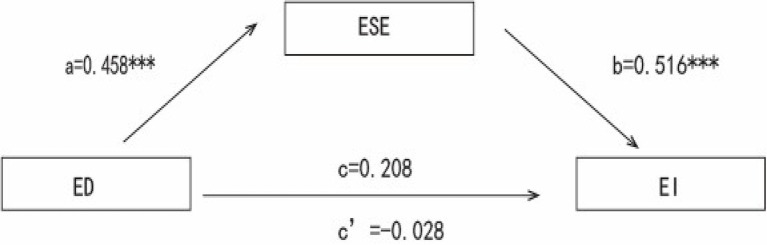
Mediating effect model. ^***^means *p* < 0.001; ED, entrepreneurship education; ESE, entrepreneurial self-efficacy; EI, entrepreneurial intention.

When testing the mediation effect model, according to Hayes (2017), the mediation process 3.4 of Model 4 in SPSS was applied. Gender, major, grade, entrepreneurial experience, entrepreneurial competition experience, and the family background of self-employment were taken as control variables. The results are shown in Supplementary Tables 4 and 5. In the absence of entrepreneurial self-efficacy, Entrepreneurship education has a significant effect on entrepreneurial intention (*β* = 0.208, *t* = 6.068, *p* < 0.001). Entrepreneurship education has a significant predictive effect on entrepreneurial self-efficacy (*β* = 0.458, *t* = 14.575, *p* < 0.001). When entrepreneurial self-efficacy was added in the analysis as a mediator, the effect of entrepreneurship education on entrepreneurial intention became insignificant (*β* = −0.028, *t* = −0.831, *p* = 0.406). However, the predictive effect of entrepreneurial self-efficacy on entrepreneurial intention is still significant (*β* = 0.516, *t* = 15.133, *p* < 0.001). Since the upper and lower limit of the mediating and total effect Bootstrap 95% confidence interval do not contain 0, the upper and lower limit of the direct effect Bootstrap 95% confidence interval contains 0. Therefore, entrepreneurial self-efficacy is a complete mediation in the relationship between entrepreneurship education and entrepreneurial intentions (indirect effect = 0.236, 95% CI = 0.177–0.301). In addition, entrepreneurial self-efficacy has suppressing effect in the relationship between entrepreneurship education and entrepreneurial intentions as the sign of the mediation effect is opposite to that of the direct effect, the total effect is suppressed. The model is shown in Figure 1.

### Testing for Moderated Mediation

The results in Supplementary Table 6 show that gender, grade, major, entrepreneurial experience, and family background have no significant moderating effects on the three paths between entrepreneurship education and entrepreneurial intention, but the moderating effect of personal entrepreneurial competition experience on the relationship between entrepreneurial self-efficacy and entrepreneurial intention is significant (*β* = −0.396, *p* < 0.001), which played a moderating role.

**Figure 2:**
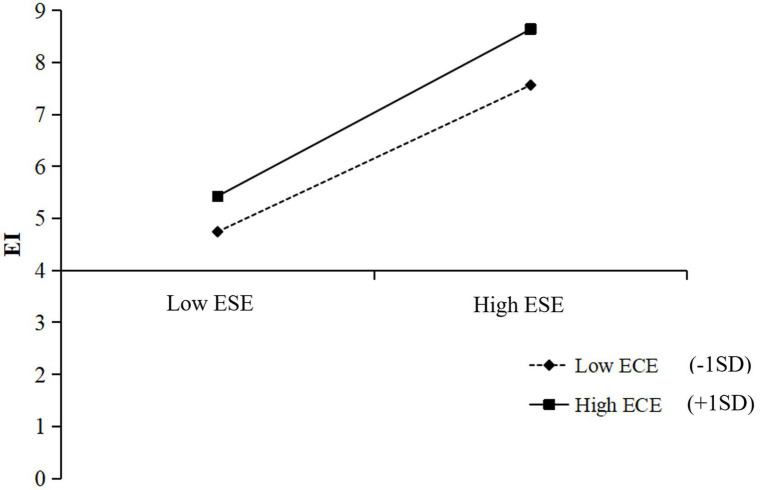
EI as a function on ESE and ECE. Simple slope analysis showed that ECE moderated the relation between ESE and EI. The function was graphed for two levels of independent variable and moderator: 1 SD above the mean and 1 SD below the mean.

Then, the entrepreneurial competition experience is used as a moderating variable, and gender, major, grade, entrepreneurial experience, and the family background of self-employment are used as control variables. The results indicate that entrepreneurial self-efficacy has a significant predictive effect on entrepreneurial intention (*b*_simple_ = 1.29, SE = 0.17, *t* = 7.36, *p* < 0.001, 95% CI [0 96, 1.58]) when the mediating variable entrepreneurial self-efficacy is put into the predictive equation of entrepreneurial intention, and the interaction of entrepreneurial self-efficacy and entrepreneurial competition experience has a significant predictive effect on entrepreneurial intention (*b*_simple_ = −0.40, SE = 0.09, *t* = −4.27, *p* < 0.001, 95% CI [−0.56, −0.22]). The further simple slope analysis (see Figure 2) showed that entrepreneurship competition experience (*b*_simple_ = 0.25, *t* = 4.64, *p* < 0.001) moderates the second half of the mediation path.

## Discussion

### Characteristics of College Students’ Entrepreneurial Intentions and Entrepreneurship Education

With regard to entrepreneurial intentions, boys’ entrepreneurial intention is significantly higher than that of girls, which is consistent with those researchers’ results, such as Kautonen et al. (2015), Raaj and Shri (2015), and Mei et al. (2016). Besides, the entrepreneurial intentions of college students with entrepreneurial experience, including entrepreneurial competition experience, are higher than those without them. This research conclusion further validates the results of Chen et al. (1998) and Mei et al. (2016), this conclusion is also in line with our common sense. Moreover, the entrepreneurial intention of students with family background of entrepreneurship is higher than those without such background, which is consistent with the results of some researchers (Henley, 2007; Gurel et al., 2010; Bignotti and Roux, 2016). This conclusion further confirms the relationship between the family background of entrepreneurship and individual entrepreneurial intentions.

For entrepreneurship education, there are significant differences in gender, entrepreneurial competition experience, and the family background of entrepreneurship. Boys’ scores are significantly lower than those of girls. One possible reason is that boys are prone to participating in practice rather than theoretical courses. Students with entrepreneurial competitions score higher than those without because they have more access to entrepreneurship education. Similarly, students with family background of self-employment score higher than those without it as actual contact with enterprises or entrepreneurship helps students improve their knowledge and skills of entrepreneurship.

### The Mediating Role of Entrepreneurial Self-Efficacy

The study confirmed a significantly positive link between entrepreneurship education and entrepreneurial intention, and entrepreneurial self-efficacy played a complete mediating role in the prediction of entrepreneurship education on entrepreneurial intention. Therefore, hypotheses H1, H2, H3 and H4 are supported. These results suggest that entrepreneurial intention of college students was more likely to be strengthened through entrepreneurial self-efficacy. The finding corroborates those of preceding studies which drew similar conclusions (Rauch and Hulsink, 2015; Udayanan, 2019; Anwar et al., 2020; Islam et al., 2020). However, some previous researchers found that entrepreneurial self-efficacy is a partial mediator between entrepreneurship education and entrepreneurial intentions (Rauch and Hulsink, 2015; Anwar et al., 2020). The finding proves that entrepreneurship education affects entrepreneurial intention mainly through entrepreneurial self-efficacy. There are still other possible mediating variables. Besides, entrepreneurial self-efficacy also has a suppressing effect on the relationship between the two, which suggests entrepreneurial self-efficacy eliminates part of the positive predictive effect of entrepreneurship education on entrepreneurial intentions. This conclusion is also consistent with common sense. For example, if students have experienced failures in the process of entrepreneurship education, their entrepreneurial efficacy is likely to decrease, which in turn weakens students’ entrepreneurial intentions.

### The Moderating Role of Entrepreneurial Competition Experience

The study further suggested that entrepreneurial competition is a moderating variable. Entrepreneurial competition experience could moderate the second half of the mediating effect of entrepreneurial self-efficacy, so the hypothesis H5 was supported. Specifically, the linkage between entrepreneurial self-efficacy and entrepreneurial intention would be stronger for individuals with high levels of entrepreneurial competition experience. The more entrepreneurial competition experience college students have, the stronger the predictive effect of entrepreneurial self-efficacy is on their entrepreneurial intentions (Bandura, 1977; Bandura et al., 2001; Carr and Sequeira, 2007; Miralles et al., 2016; Jiao et al., 2021). Competition experience has a very good promotion effect on entrepreneurial intention of college students, which exists regardless of the level of entrepreneurial self-efficacy. The reason may lie in their more acquaintance with the entrepreneurial process and greater involvement in entrepreneurial competitions. With their entrepreneurial knowledge and practical skills improved, their entrepreneurial self-efficacy will probably increase. If they finally win the competition, it is even more helpful to enhance their entrepreneurial self-efficacy. As students’ entrepreneurial self-efficacy accelerates, so do their entrepreneurial intentions.

## Implications

The current study has explored the internal mechanism of entrepreneurship education on entrepreneurial intentions in depth through the mediator of entrepreneurial self-efficacy and the moderator of entrepreneurial competition experience. To some extent, the findings have enriched and improved the theoretical system of entrepreneurship education and entrepreneurial intentions model. Specifically, this study shows that entrepreneurial self-efficacy plays a complete mediating role between entrepreneurship education and entrepreneurial intentions. Hence, entrepreneurial education could enhance entrepreneurial self-efficacy by offering necessary entrepreneurial knowledge and techniques, which could further affect entrepreneurial intention (Ali, 2013). In addition, this study investigates the moderating role of entrepreneurial competition experience. Some previous studies focused on the moderating role of creativity and gender on entrepreneurial intention (Ryu and Kim, 2020; Shi et al., 2020) or regional social capital in the entrepreneurial intention-behavior link (Weiss et al., 2019). A few studies reveal that entrepreneurial competition experience also plays a significant moderating role between entrepreneurial self-efficacy and entrepreneurial intentions.

As far as the practical implications are concerned, first, it is necessary to take targeted educational measures to improve girls’ entrepreneurial self-efficacy so as to promote their entrepreneurial intentions in the light of gender difference in entrepreneurial intentions. In addition, in consideration of the impact of personal entrepreneurial experience and entrepreneurial competition experience on entrepreneurial intentions, we found that entrepreneurship education affect entrepreneurial intention mainly through entrepreneurial self-efficacy. Therefore, entrepreneurship education could focus more on creating practical opportunities and better entrepreneurial practice platforms to help students accumulate experience. Teachers should encourage college students to participate in entrepreneurial competitions more frequently not only to raise their self-confidence and courage but also to heighten their entrepreneurial intentions as prior experience is a valuable resource regardless of success or failure.

## Limitations and Future Research

Several limitations of this study should be noted. First of all, this study uses cross-sectional study, so it cannot really fully explore the mechanism of entrepreneurship education on entrepreneurial intentions. A longitudinal study would be better to further examine the development of college students’ entrepreneurial intentions with age. The entrepreneurial intentions of graduated students have not been investigated and tracked. Besides, as the research is a questionnaire survey, the quality of the data may be affected by respondents’ social desirability. Moreover, the sample of the study is also subject to geography. Future studies are expected to cover a diverse group of potential entrepreneurs and increase representative samples. Finally, this research analyzes the entrepreneurial intentions of college students from the perspectives of entrepreneurship education and entrepreneurial self-efficacy. These factors are representative variables of entrepreneurial actions. However, there are still some other variables that affect entrepreneurial intentions, such as entrepreneurial motivation and entrepreneurial spirit. Besides, other demographic variables of an individual, such as growing environment, work, and non-work environment, may affect an individual’s entrepreneurial intention to a certain extent. Therefore, in future research, we can try to incorporate other social characteristics of individuals into the entrepreneurial intention model to do more in-depth research.

## Data Availability Statement

The raw data supporting the conclusions of this article will be made available by the authors, without undue reservation.

## Ethics Statement

The studies involving human participants were reviewed and approved by Wenzhou Medical University Ethics Committee. The patients/participants provided their written informed consent to participate in this study. Written informed consent was obtained from the individual(s) for the publication of any potentially identifiable images or data included in this article.

## Author Contributions

All authors listed have made a substantial, direct and intellectual contribution to the work, and approved it for publication.

## Conflict of Interest

The authors declare that the research was conducted in the absence of any commercial or financial relationships that could be construed as a potential conflict of interest.

## Publisher’s Note

All claims expressed in this article are solely those of the authors and do not necessarily represent those of their affiliated organizations, or those of the publisher, the editors and the reviewers. Any product that may be evaluated in this article, or claim that may be made by its manufacturer, is not guaranteed or endorsed by the publisher.
