# Supplementary material for: Entrepreneurship Education and Entrepreneurial Intentions of College Students: The Mediating Role of Entrepreneurial Self-Efficacy and the Moderating Role of Entrepreneurial Competition Experience
Source: Front Psychol. 2022 Jan 6;12:727826. doi: 10.3389/fpsyg.2021.727826 (PMC8770817; doi:10.3389/fpsyg.2021.727826)
Supplement: Supplementary file 1 [file Table_1.docx]

**Table 1** Statistics of sample characteristics of categorical variables (dependent variable: entrepreneurial intention)

| Variables | Classification | N | M | SD | SE | 95% CI | |
| --- | --- | --- | --- | --- | --- | --- | --- |
|  |  |  |  |  |  | Lower | Upper |
| Gender  Major  Grade  EE  ECE  FS | Male  Female  Medicine  S & E  Eco-management  Humanities  Other  Freshman  Sophomore  Junior  Senior  G & A  Yes  No  Yes  No  Yes  No | 236  568  274  147  232  112  39  379  86  269  61  9  59  745  103  701  162  642 | 2.67  2.39  2.47  2.31  2.56  2.47  2.55  2.46  2.60  2.44  2.56  2.13  3.10  2.42  2.77  2.43  2.72  2.41 | .92  .84  .83  .84  .91  .92  .82  .82  .87  .90  1.02  1.17  .86  .85  1.06  .83  .96  .84 | .06  .04  .05  .07  .06  .09  .13  .04  .09  .05  .13  .39  .11  .03  .10  .03  .08  .03 | 2.558  2.316  2.373  2.170  2.440  2.299  2.2869  2.378  2.410  2.329  2.296  1.224  2.873  2.360  2.564  2.365  2.575  2.342 | 2.792  2.455  2.571  2.442  2.677  2.642  2.820  2.543  2.783  2.544  2.819  3.030  3.321  2.482  2.979  2.488  2.872  2.472 |

Note: S&E, Science and Engineering; G&A, Graduate and above; EE, entrepreneurial experience; ECE, entrepreneurial competition experience; FS, family of self-employment.

**Table 2** Statistics of sample characteristics of categorical variables (dependent variable: entrepreneurship education)

| Variables | Classification | N | M | SD | SE | 95% CI | | |
| --- | --- | --- | --- | --- | --- | --- | --- | --- |
|  |  |  |  |  |  | Lower | | Upper |
| Gender  Major  Grade  EE  ECE  FS | Male  Female  Medicine  S & E  Eco-management  Humanities  Other  Freshman  Sophomore  Junior  Senior  G & A  Yes  No  Yes  No  Yes  No | 236  568  274  147  232  112  39  379  86  269  61  9  59  745  103  701  162  642 | 3.22  3.42  3.23  3.37  3.46  3.41  3.43  3.38  3.44  3.29  3.43  3.23  3.50  3.35  3.75  3.30  3.52  3.32 | .88  .78  .76  .74  .87  .87  .84  .72  .85  .85  1.09  .79  .81  .81  .87  .79  .86  .79 | .06  .03  .05  .06  .06  .08  .13  .04  .09  .05  .14  .26  .10  .03  .09  .03  .07  .03 | 3.107  3.353  3.142  3.250  3.351  3.250  3.162  3.308  3.260  3.188  3.153  2.624  3.287  3.2899  3.5794  3.2435  3.3875  3.2568 | 3.332  3.481  3.323  3.492  3.576  3.574  3.704  3.453  3.626  3.392  3.711  3.845  3.707  3.407  3.920  3.360  3.654  3.380 | |

Note: S&E, Science and Engineering; G&A, Graduate and above; EE, entrepreneurial experience; ECE, entrepreneurial competition experience; FS, family of self-employment.

**Table 3** The correlation coefficient between entrepreneurship education, entrepreneurial self-efficacy and entrepreneurial intention

| Variables M±SD 1 2 3 4 5 6 | | | | | |
| --- | --- | --- | --- | --- | --- |
| 1 EE  2 ECE  3 FS  4 ED  5 ESE  6 EI | 1.93±0.26 1.00 | | | | |
|  | 1.87±0.33 0.12**  1.80±0.40 0.13**  3.36±0.81 -0.05  3.18±0.74 -0.16**  2.47±0.87 -0.20** | 1.00 | | | |
|  |  | 0.04  -0.19**  -0.15**  -0.13** | 1.00 | | |
|  |  |  | -0.10**  -0.18**  -0.15** | 1.00 | |
|  |  |  |  | 0.47** 1.00  0.22** 0.54** | 1.00 |

Note: ** means p<0.01; EE, entrepreneurial experience; ECE, entrepreneurial competition experience; FS, family of self-employment; ED, entrepreneurship education; ESE, entrepreneurial self-efficacy; EI, entrepreneurial intention.

**Table 4** Mediation model test of entrepreneurial self-efficacy

| Predictor Variables | Outcome Variables | | | | | |
| --- | --- | --- | --- | --- | --- | --- |
|  | EI | | ESE | | EI | |
|  | β | t | β | t | β | t |
| Gender | -0.364 | -4.945*** | -0.203 | -3.009** | -0.259 | -3.972*** |
| Major | 0.013 | 0.439 | -0.053 | -1.967* | 0.040 | 1.549 |
| Grade | -0.012 | -0.381 | 0.013 | 0.446 | -0.019 | -0.671 |
| EE | -0.622 | -4.808*** | -0.432 | -3.646*** | -0.400 | -3.469*** |
| ECE | -0.218 | -2.140* | -0.156 | -1.669 | -0.138 | -1.529 |
| FS | -0.236 | -2.815** | -0.294 | -3.825*** | -0.084 | -1.131 |
| ESE |  |  |  |  | 0.516 | 15.133*** |
| ED | 0.208 | 6.068*** | 0.458 | 14.575*** | -0.028 | -0.831 |
| R | 0.358 | | 0.516 | | 0.568 | |
| R2 | 0.128 | | 0.267 | | 0.323 | |
| F | 16.710*** | | 41.337*** | | 47.436*** | |

Note: *** means p<0.001; ** means p<0.01; * means p<0.05; all variables in the regression equation are standardized and then brought into analysis. EE, entrepreneurial experience; ECE, entrepreneurial competition experience; FS, family of self-employment; ED, entrepreneurship education; ESE, entrepreneurial self-efficacy; EI, entrepreneurial intention.

**Table 5** Breakdown table of mediating, direct and total effect

|  | ES | Boot SE | Boot CI LL | Boot CI UL | ER |
| --- | --- | --- | --- | --- | --- |
| Mediation | 0.236 | 0.032 | 0.177 | 0.301 | 113.462% |
| Direct | -0.028 | 0.040 | -0.108 | 0.051 | -13.462% |
| Total | 0.208 | 0.045 | 0.134 | 0.309 |  |

Note: The bias-corrected percentile method is used to estimate Bootstrap's standard error and 95% confidence interval. ES, effect size; ER, effect ratio.

**Table 6** Test of moderated mediation effects

|  | E1 DV:ESE | | | | E2 DV：EI | | | |
| --- | --- | --- | --- | --- | --- | --- | --- | --- |
|  | b | SE | t | 95%CI | b | SE | t | 95%CI |
| ED | 0.22 | 0.17 | 1.33 | [-0.19,0.70] | -0.13 | 0.17 | -0.78 | [-0.49,0.21] |
| ECE | -0.26 | 0.10 | -2.53* | [-0.45,-0.05] | -0.06 | 0.10 | -0.57 | [-0.23,0.11] |
| ED×ECE | 0.13 | 0.09 | 1.44 | [-0.13,0.35] | 0.05 | 0.09 | 0.54 | [-0.13,0.25] |
| ESE |  |  |  |  | 1.29 | 0.17 | 7.36*** | [0.96,1.58] |
| ESE×ECE |  |  |  |  | -0.40 | 0.09 | -4.27*** | [-0.56,-0.22] |
| Control variables： | | | | | | | | |
| Gender | -0.07 | 0.06 | -1.25 | [-0.18,0.04] | -0.27 | 0.07 | -4.16*** | [-0.40,-0.14] |
| Major | -0.01 | 0.02 | -0.43 | [-0.05,0.03] | 0.03 | 0.03 | 1.03 | [-0.02,0.08] |
| Grade | -0.01 | 0.03 | -0.30 | [-0.06,0.05] | -0.02 | 0.03 | -0.58 | [-0.08,0.05] |
| EE | -0.38 | 0.10 | -3.87*** | [-0.58,-0.18] | -0.59 | 0.11 | -5.16*** | [-0.81,-0.37] |
| FS | -0.30 | 0.07 | -4.64*** | [-0.43,-0.16] | -0.25 | 0.08 | -3.37** | [-0.40,-0.10] |
| F | 77.55*** | 72.07*** |  |  |  |  |  |  |
| R^2^ | 0.23 | 0.31 |  |  |  |  |  |  |

Note: Unstandardized regression coefficients are obtained using Bootstrap method. EE, entrepreneurial experience; ECE, entrepreneurial competition experience; FS, family of self-employment; ED, entrepreneurship education; ESE, entrepreneurial self-efficacy; EI, entrepreneurial intention; DV, dependent variable.
